# Supplementary material for: Metabolic and behavioral alterations associated with viral vector-mediated toxicity in the paraventricular hypothalamic nucleus
Source: Biosci Rep. 2024 Jan 25;44(1):BSR20231846. doi: 10.1042/BSR20231846 (PMC10830444; doi:10.1042/BSR20231846)
Supplement: Supplementary Figures S1-S5 [file BSR-2023-1846_supp.pdf]

Supplementary Information

Metabolic and behavioral alterations associated with viral vector-mediated toxicity in the paraventricular hypothalamic nucleus

Rohan Savani[1,2], Erin Park[1,2], Nidhi Busannagari[1,2], Yi Lu[1], Hyokjoon Kwon[3], Le Wang[1, *],
Zhiping P. Pang[1, *]

[1]The Child Health Institute of New Jersey, Robert Wood Johnson Medical School, Rutgers, The State University of New Jersey, New Brunswick, NJ, USA

[2]Department of Cell Biology and Neuroscience, Undergraduate Program, Rutgers, The State University of New Jersey, New Brunswick, NJ, USA

[3]Department of Medicine, Rutgers-Robert Wood Johnson Medical School, New Brunswick, NJ, 08901, USA

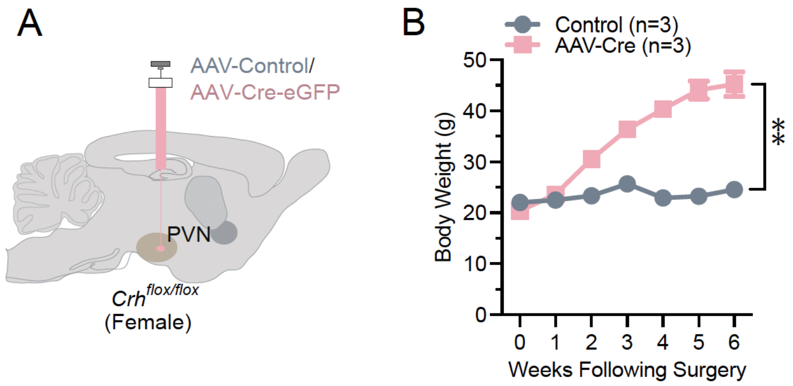

Figure S1: AAV-Cre expression in the PVN of female *Crh*$^{f/f}$ mice recapitulates body weight increases. (A) Experimental paradigm for virus delivery. **(B)** Body weight increases following AAV-Cre injection (two-way ANOVA, main effect of Group: F (1, 4) = 59.08, p = 0.0015; main effect of Time: F (1.782, 7.129) = 77.74, p < 0.0001; interaction between Group and Time: F (6, 24) = 58.41, p < 0.0001). Sample sizes are as shown on graphs (n = 3 mice per group). Data are presented as mean ± SEM.

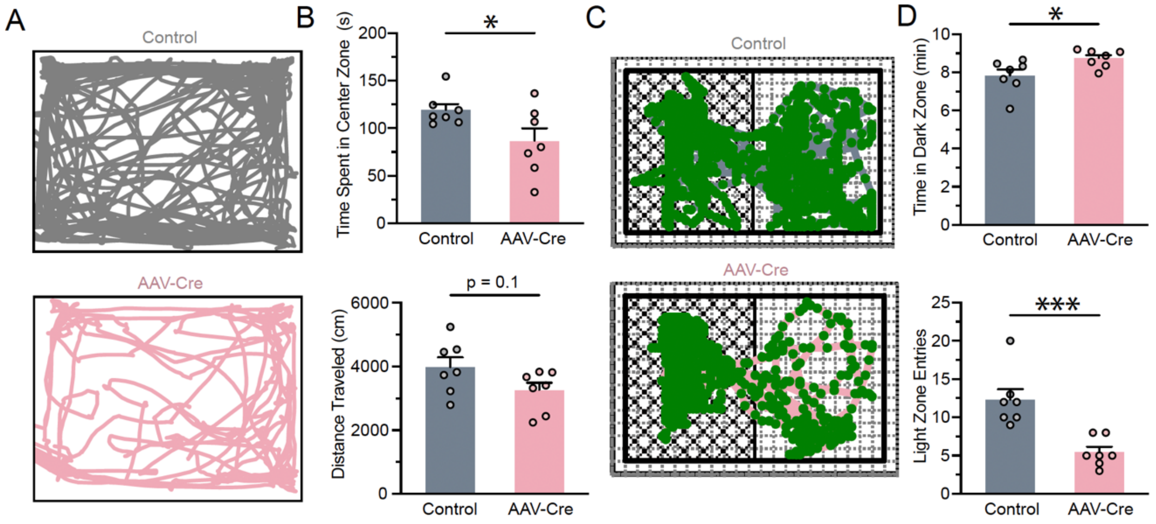

Figure S2: ***Crh***$^{f/f}$ **mice expressing AAV-Cre in PVN display elevated anxiety-like behaviors.** **(A)** Representative trajectories of AAV-GFP- (top) and AAV-Cre-injected (bottom) male mice during the open field test. **(B)** AAV-Cre-injected mice spent less time in the center zone of the open field test (t (12) = 2.181, p = 0.0498), and changes in distance traveled were not significant (t (12) = 1.811, p = 0.0952) during the open field test. **(C)** Representative trajectories of both groups during the light-dark box test. **(D)** Time spent in the dark zone increased (t (12) = 2.416, p = 0.0326), and entries into the light zone decreased (Mann-Whitney test, U = 0, p = 0.0006) during the light-dark box test. Sample sizes are as shown on graphs (n = 7 mice per group). Data are presented as individual points and mean + SEM.

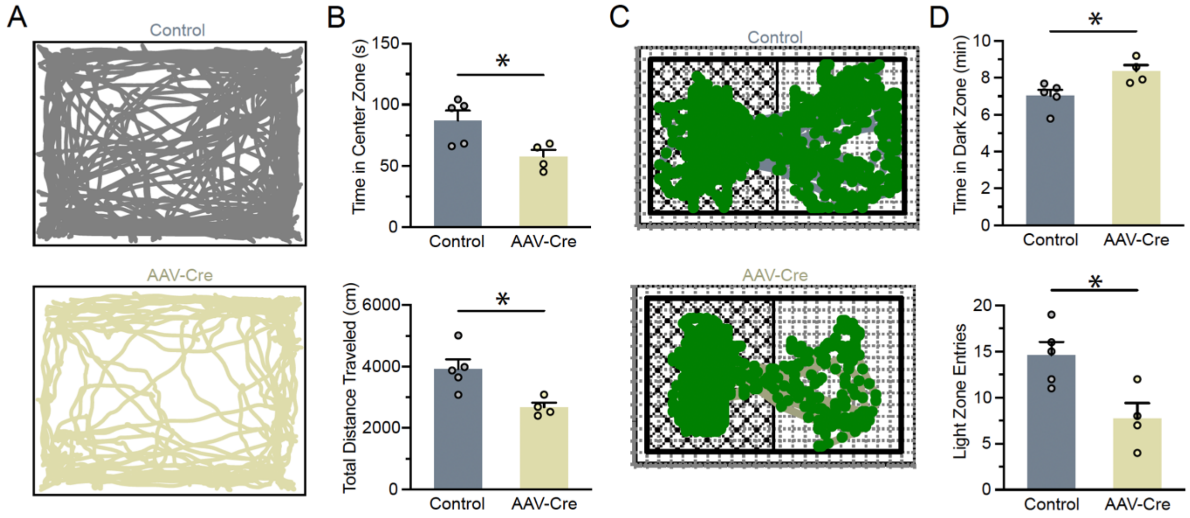

Figure S3. Wild-type male mice injected with AAV-Cre in PVN exhibit increased anxiety-like behaviors. (A) Representative traces of wild-type mice during the open field test. **(B)** During the open field test, time spent in the center zone decreased (t (7) = 2.817, p = 0.0259), and total distance traveled decreased (t (7) = 3.275, p = 0.0136). **(C)** Representative movement during the light-dark box test for both groups. **(D)** During the light-dark box test, time spent in the dark zone increased (t (7) = 2.798, p = 0.0266), and entries into the light zone decreased (t (7) = 3.142, p = 0.0163). Sample sizes are as shown on graphs (n = 5 AAV-GFP-injected mice, n = 4 AAV-Cre-injected mice). Data are presented as individual points and mean + SEM.

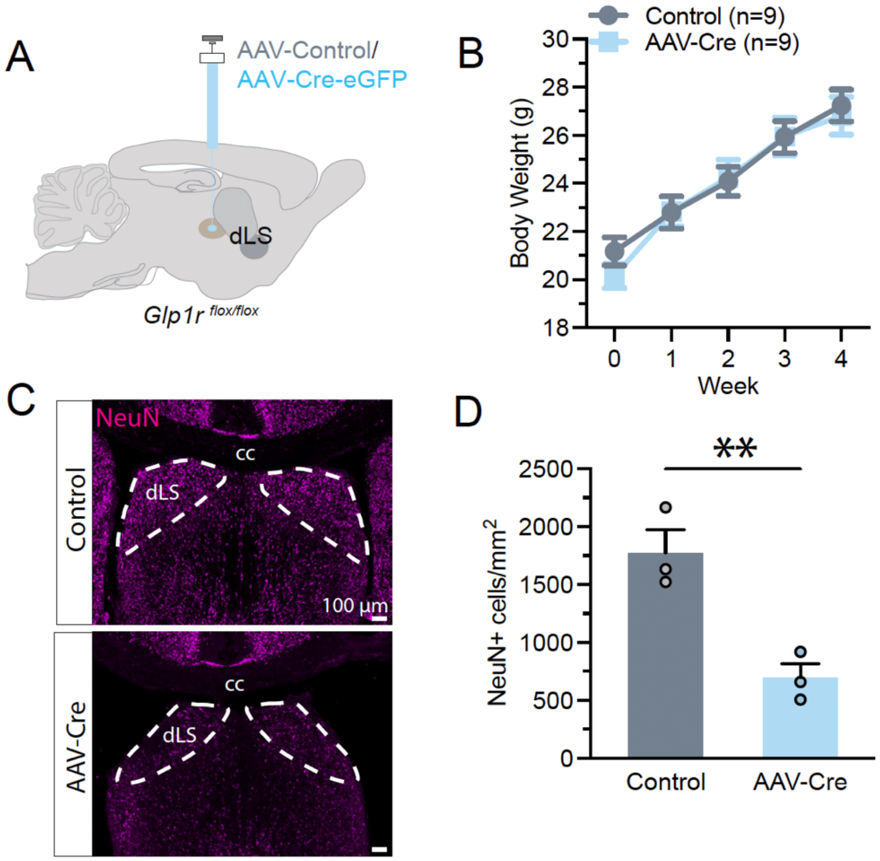

Figure S4. AAV-Cre expression in the dLS of male *Glp1r*$^{f/f}$ mice results in cell death without metabolic deficits. (A) Experimental paradigm for virus delivery. **(B)** Body weight was unchanged (two-way ANOVA, main effect of Group: F (1, 16) = 0.07282, p = 0.7907; main effect of Time: F (1.826, 29.22) = 364.6, p < 0.0001; interaction between Group and Time: F (4, 64) = 3.406, p = 0.0138). **(C)** Representative images of neuronal nuclei in the dLS after AAV-GFP or AAV-Cre injection. **(D)** Neuron density decreased in the dLS after AAV-Cre expression (t (4) = 4.631, p = 0.0098). Sample sizes are as shown on graphs: **(B)** n = 9 mice per group; **(D)** n = 3 mice per group. Data are presented as individual points and mean ± or + SEM. cc: corpus callosum.

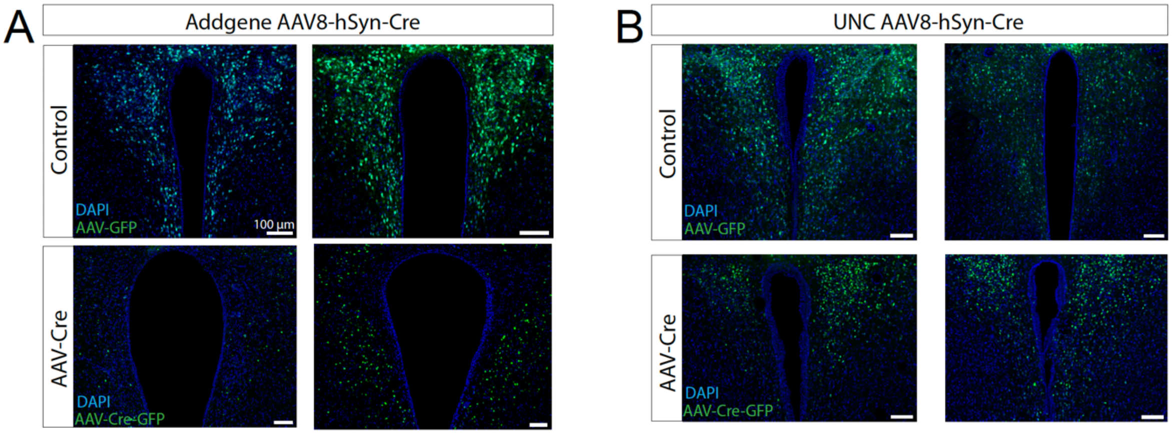

Figure S5: AAV-Cre-GFP Expression with AAV-Cre and AAV-GFP in PVN. (A) Examples of expression of AAV-GFP (control) or AAV-Cre-GFP in *Crh-flox* mice correspond to Figure 2. **(B)** Examples of expression in *Crh-flox* mice injected with the UNC Vector Core-sourced AAV-Cre correspond to Figure 4.
